# Percutaneous Nephrolithotomy Combined Antegrade Flexible Ureteroscope for Complete Staghorn Stones: A Case Report of a New Concept of Stone Surgery

**DOI:** 10.3390/medicina59010035

**Published:** 2022-12-23

**Authors:** Szu-Ying Pan, Chi-Ping Huang, Wen-Chi Chen, Yung-Hsiang Chen, Eric Chieh-Lung Chou

**Affiliations:** 1 Department of Urology, China Medical University Hospital, Taichung 40447, Taiwan; 2 School of Medicine, College of Medicine, China Medical University, Taichung 40402, Taiwan; 3 Graduate Institute of Integrated Medicine, College of Chinese Medicine, China Medical University, Taichung 40402, Taiwan; 4 Department of Psychology, College of Medical and Health Science, Asia University, Taichung 41354, Taiwan

**Keywords:** staghorn stone, percutaneous nephrolithotomy, endoscopic combined intrarenal surgery, flexible ureteroscopy

## Abstract

Percutaneous nephrolithotomy (PCNL) is the treatment of choice for staghorn stones. However, residual stones in calyces remain a challenge due to the limited angle which makes the approach difficult. The new operative technique of endoscopic combined intrarenal surgery (ECIRS), which integrates the advantages of PCNL and retrograde intrarenal surgery (RIRS), was developed to overcome this difficulty. However, two experienced urologists are required to perform ECIRS, and the patient has to be placed in the Galdakao-modified supine Valdivia position or modified prone split-leg position which cannot be achieved in the elderly or patients with ankylosing arthritis, as it may cause harm due to abnormal traction of the joints. In addition, it is difficult for surgeons to create an ideal access tract to perform PCNL in this position. We report the case of a 72-year-old female patient with left staghorn stone. We performed RIRS first and then placed the patient in the decubitus position for PCNL with antegrade flexible ureteroscopy. This method allows patients to be placed in an easier position, with the use of flexible ureteroscopy through a nephroscope to find previously unreachable stones. Moreover, in addition to the more comfortable position both for surgeons and patients, this procedure can also deal with large complex renal stones as with ECIRS. We also created a brand-new definition for stone clearance rate, namely, stone reduction efficiency (SRE). There was a high stone reduction efficiency of 12.64 (mm^2^/min) in our patient, and no complications occurred. We suggest that this procedure is an ideal alternative treatment for a huge staghorn stone instead of PCNL or ECIRS.

## 1. Introduction

Percutaneous nephrolithotomy (PCNL) is the first choice for treating partial and complete staghorn renal stones [1,2], and the latest European Association of Urology (EAU) 2021 guidelines recommend PCNL or retrograde intrarenal surgery (RIRS) for kidney stones > 20 mm [3]. Articles comparing stone clearance rates between flexible ureteroscopy (fURS) and PCNL for complex renal stones have also reported that the latter method outperforms the former for larger renal stones > 2 cm in size [4]. A new surgical technique combining both methods called endoscopic combined intrarenal surgery (ECIRS) has been developed [5] and demonstrated to result in better outcomes than traditional surgery for complex kidney stones without ureteral stones. However, there are many drawbacks with ECIRS including double endourologic armamentarium, and kidney hypermobility due to the Galdakao modified supine Valdivia (GMSV) position [6]. We report a 72-year-old female patient with left flank pain caused by a left complete staghorn stone. We conducted PCNL combining antegrade fURS with a high-power laser and achieved good results.

## 2. Case Presentation

A 72-year-old female with a medical history of hypertension, poorly controlled diabetes mellitus, chronic kidney disease and refractory urinary tract infection presented with severe flank pain for over 1 year. She had visited two urologists; the first recommended ECIRS, and the other referred her to a medical center due to a lack of two urologists and double endourologic armamentarium. She then came to our hospital for a third opinion because she could not afford the cost of ECIRS and difficulty in being placed in the GMSV position.

**Figure 1:**
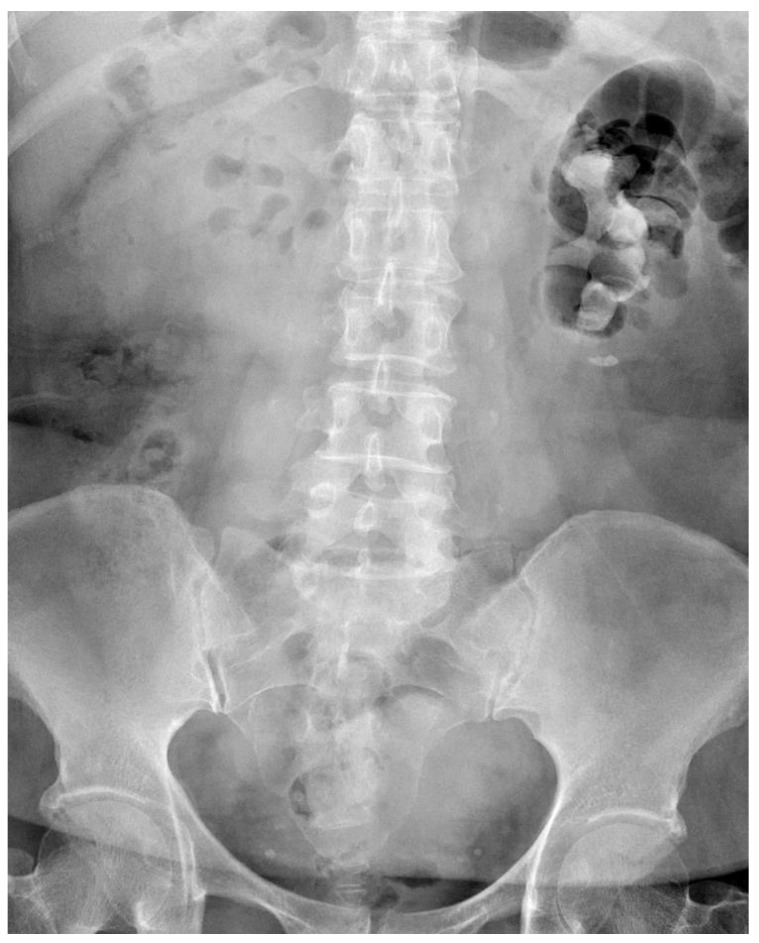
Preoperative findings of a kidney, ureter and bladder radiograph that were suggestive of a large staghorn stone about 7 cm in size.

Laboratory studies including complete blood count and electrolyte profiles were all within normal ranges. However, blood sugar, glomerular filtration rate (52 mL/min/1.73 m^2^) and urine analysis (proteinuria and pyuria) were abnormal. A kidney ureter bladder (KUB) radiograph revealed a stone occupying all three calyces and renal pelvis, with a maximum length of 7 cm (Figure 1). Following PCN insertion done by a radiologist, computed tomography showed that the PCN tract had been created through the middle calyx of the left kidney.

**Figure 2:**
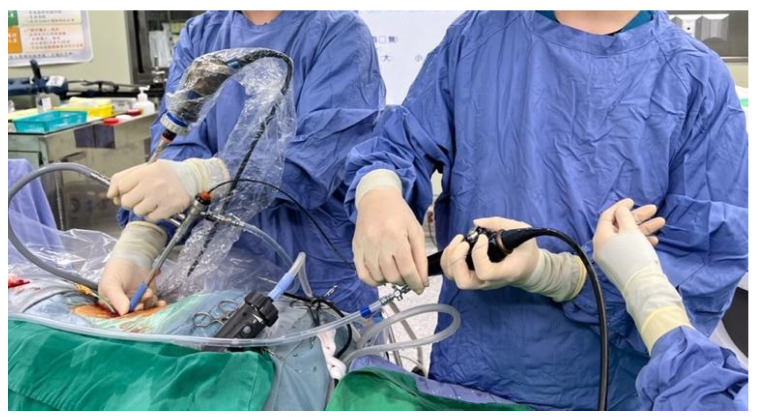
Operational assistance (**left** side) held the nephroscope, and the experienced endourologist (**right** side) performed flexible ureteroscopy.

**Figure 3:**
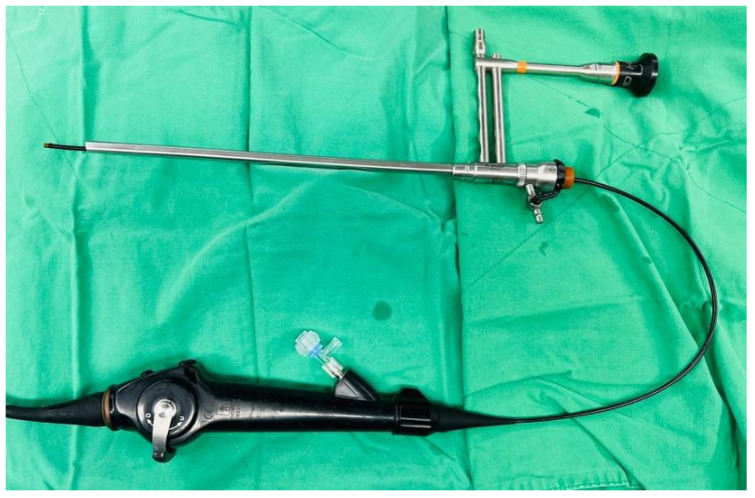
8 Fr flexible ureteroscope was placed into the working tract of a nephroscope.

Taking her economic conditions and complicated underlying diseases into consideration, it was decided that a second operation would be too risky if we failed to decrease most of her stone burden this time. Hence, we formulated a modified way of performing the operation. First, the patient was placed in the lithotomy position, and left RIRS was performed with an 11 Fr ureteral access sheath for the stone at the ureteropelvic junction and renal pelvis, and the stone was fragmented using a 272-nm high-power holmium-YAG laser. After retrograde indwelling of a double-J catheter, her position was changed to the left lateral decubitus with mild flank flexion. An axillary roll was placed between her chest wall and the bed to avoid brachial plexus compression. The PCNL tract was made with a PCN catheter with cold knife urethrotomy to allow a 24 Fr Amplatz sheath to reach the staghorn stone. We performed PCNL through a 20.5 Fr nephroscope and disintegrated the visible stone with an ultrasonic lithotriptor. We then started the modified procedure. We used the nephroscope to locate the upper part of the middle calx and upper calyx where we thought residual stones may exist. Under assistance (Figure 2), the operator inserted an 8 Fr fURS into the working channel of the nephroscope (Figure 3). The high-power holmium laser was then used to fragment visible stones as far as possible.

**Figure 4:**
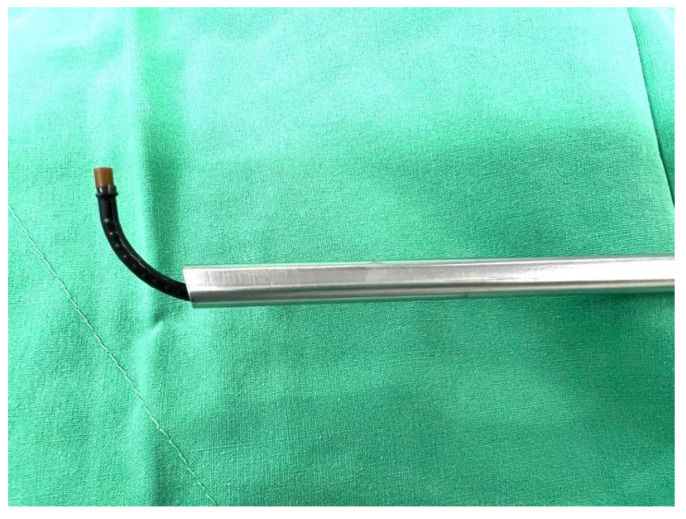
Flexible ureteroscope working extent.

This method increased the reach of the rigid nephroscope (Figure 4) without causing much iatrogenic tissue injury. A 20 Fr nephrostomy was inserted for post-operative drainage. The total operation time was 1 h and 27 min, and the estimated blood loss was 100 mL. No complications occurred after the surgery, and the nephrostomy tube was removed 3 days after the operation. The total hospital course was 5 days.

**Figure 5:**
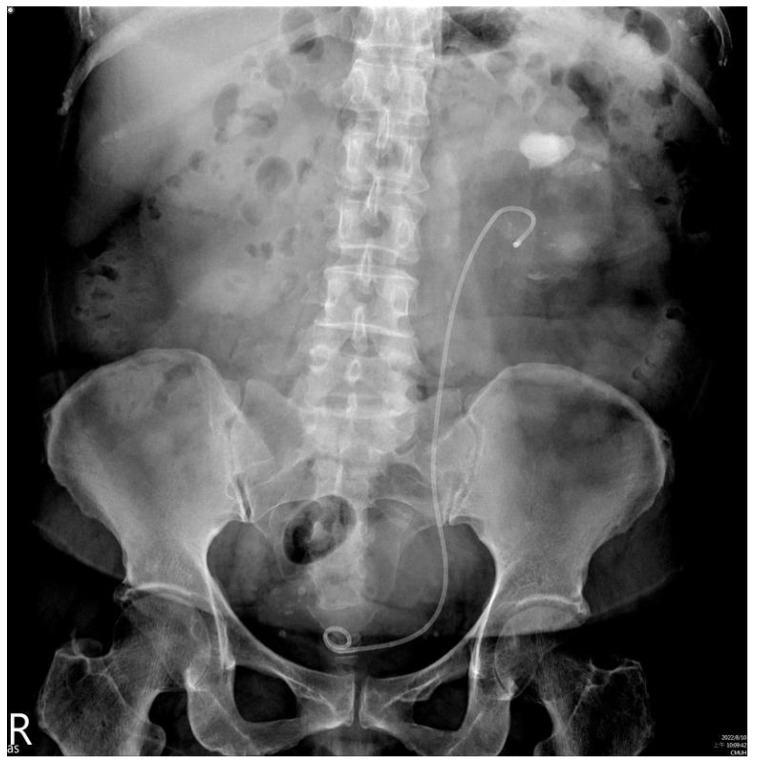
Postoperative findings of a kidney, ureter and bladder radiograph suggest a residual stone about 24 mm in size.

A follow-up KUB (Figure 5) was taken 2 months after the operation, and one residual stone about 24 mm in size and stone sands were found. The stone reduction rate was 76.7%. Laboratory data measured during the same clinic visit showed a decline in creatinine from 1.32 to 1.09 mg/dL. She is currently receiving regular follow-up for renal function, KUB, and renal echo every 6 months to 1 year, and she has not reported flank discomfort.

## 3. Discussion

Staghorn stones are always a challenge for urologists. Unlike ureteral stones, staghorn calculi cause proportionally more pain, are more commonly noted in females, and typically present with an indolent clinical course [7]. Deferral of treatment may lead to renal failure or urosepsis [8]. However, due to its low stone clearance rate by PCNL, many new or modified methods, and even open surgery still remain an option [9]. However, the risk is always the most important consideration for surgeons. Therefore, many review articles and meta-analyses have been published comparing PCNL with other endoscopic methods of surgery such as ECIRS, which has a better stone clearance rate, fewer complications, and less blood transfusion compared with PCNL [10]. However, due to its cost, operation position and need for two groups of urologists, not many patients are ultimately suitable for ECIRS.

In our case, we performed RIRS with a laser first for the ureteropelvic junction and renal pelvis parts of the staghorn stone, and then shifted to PCNL combined with antegrade fURS. The combination of traditional ultrasonic lithotripsy with a new generation of flexible scope and high-energy laser successfully overcame the main limitation of a rigid scope. We also found, by a using nephroscope, that the operator can target the stone accurately without x ray exposure and have a clearer operation view because of the irrigation channel of nephroscope especially when there is bleeding. The stone reduction rate was 76.7%. Few recent studies have reported on the use of ECIRS for staghorn stones, and only one paper has compared ECIRS with minimally invasive PCNL for complex renal stones [11]. Although stone clearance is important, we think that operation time is a vital factor that every surgeon should never ignore. Hence, we create a brand-new definition for stone clearance that is stone reduction efficiency (SRE) which defined as stone burden (mm^2^) divided by operation time (minutes). The stone reduction efficiency calculated for their study was 8.1 (mm^2^/min), compared to 12.64 (mm^2^/min) in our case. Therefore, our modified surgical technique for stone reduction was 1.56 times more efficient than in the previous case. Moreover, only 15% of the patients had staghorn stones in the ECIRS group, and if they had included more patients with staghorn stones in the ECIRS group the stone reduction efficiency for the whole staghorn stone group would definitely be lower than 8.1 (mm^2^/min). For high-risk patients, greater stone fragmentation efficiency would reduce the operation time and definitely the risk that patients take.

Although our patient was not stone-free due to our lack of experience and of real-time X-ray technology, the stone reduction rate was good and stone reduction efficiency was superior to recent data. We believe that with the assistance of a C-arm and more experience, this procedure will be an option for complete staghorn stones in the future.

In conclusion, PCNL combined with antegrade fURS for complete staghorn stones is a safe, feasible and efficient technique. It can be a good treatment option for high-risk patients with partial/complete staghorn stones or large renal stones.

## Data Availability

All of the data are available upon request to the corresponding author.
